# VLP-based indirect ELISA enables reliable sero-diagnosis and epidemiological monitoring of duck circovirus

**DOI:** 10.3389/fvets.2026.1735016

**Published:** 2026-02-19

**Authors:** Shihua Qiu, Fangchao Guo, Lingyan Zhao, Zhaozhen Zhong, Yuancheng Wang

**Affiliations:** 1 School of Veterinary Medicine, Jiangsu Agri-Animal Husbandry Vocational College, Taizhou, China; 2 Jiangsu University of Science and Technology, Zhenjiang, China; 3 Fuxin Liuhe Agriculture and Animal Husbandry Co., Ltd. Poultry Branch, Liaoning, China

**Keywords:** diagnostic assay, duck circovirus, epidemiology, indirect ELISA, serodiagnosis, virus-like particle

## Abstract

**Introduction:**

Duck circovirus (DuCV) is an emerging immunosuppressive virus that causes growth retardation, feather loss, and increased susceptibility to secondary infections in ducks, leading to significant economic losses in the duck industry. However, existing serological assays for DuCV detection often lack accuracy and reproducibility due to antigen instability, limiting effective disease surveillance.

**Methods:**

To develop a more reliable diagnostic tool, the full-length capsid (Cap) gene of DuCV-2 was codon-optimized, expressed in *Escherichia coli*, purified under denaturing conditions, and refolded to self-assemble into virus-like particles (VLPs). The morphology of the assembled VLPs (~15 nm) was confirmed by transmission electron microscopy, and their immunogenicity was evaluated in ducks. Based on these VLPs, an indirect enzyme-linked immunosorbent assay (iELISA) was established and optimized.

**Results:**

The VLPs elicited stronger antibody responses than Cap monomers at equivalent doses, confirming their superior antigenicity. The optimized VLP-based iELISA (250 ng/well coating concentration, 1,200 serum dilution) exhibited high sensitivity (detectable up to 1:6400 dilution), strong specificity (no cross-reactivity with other avian pathogens), and good repeatability (CV < 5%). Application of the assay to 290 field duck sera collected in Jiangsu Province (2022–2024) revealed a 19.96% positivity rate, showing a gradual yearly increase.

**Discussion:**

This study developed a highly specific, sensitive, and reproducible VLP-based iELISA for DuCV antibody detection. The method provides a practical tool for large-scale epidemiological surveillance and vaccine evaluation, and the VLP-based diagnostic strategy offers a universal framework for serological assays of other avian circoviruses.

## 1 Introduction

The meat duck industry represents a critical economic pillar of global poultry production, where the health and productivity of ducks directly determine profitability. In recent years, duck circovirus (DuCV) has emerged as an important constraint to sustainable duck farming (1). Beyond typical clinical signs such as growth retardation and feather loss, DuCV causes severe damage to immune organs (e.g., thymus and bursa of Fabricius) (2, 3). This immune suppression increases susceptibility to secondary infections with pathogens such as duck enteritis virus (DEV) and duck hepatitis virus (DHV), resulting in mixed infections and substantial economic losses (4–7). Therefore, developing an efficient and accurate diagnostic tool for DuCV is urgently needed.

DuCV belongs to the family *Circoviridae*, characterized as a non-enveloped, single-stranded circular DNA virus with icosahedral particles (≈15–16 nm in diameter) (8). Since its first isolation and identification in Mulard duck flocks in Germany in 2003, DuCV has spread globally, showing increasing genetic diversity (8, 9). To date, three distinct genotypes (DuCV-1, DuCV-2, and DuCV-3) have been confirmed (10–12). The viral genome (≈1.9 kb) contains three major open reading frames (ORF1, ORF2, ORF3), among which ORF2 encodes the capsid protein (Cap), the primary structural and immunogenic protein of DuCV (13). Cap stimulates the host to produce specific neutralizing antibodies, making it a core target for developing DuCV serological diagnostics and vaccines (14, 15).

Despite progress in molecular detection, clinical diagnosis of DuCV remains challenging. DuCV infections are often accompanied by co-infections with other duck pathogens (e.g., goose parvovirus, GPV; duck tembusu virus, DTMUV), leading to overlapping clinical symptoms that render accurate diagnosis via pathological observation alone infeasible (1, 16–18). Molecular assays such as PCR and real-time PCR offer high sensitivity but require specialized equipment and are prone to false positives due to nucleic acid contamination. Moreover, previous ELISAs based on recombinant Cap monomers suffer from several drawbacks. Cap proteins are prone to denaturation and misfolding during purification, leading to the loss of conformational epitopes, increased non-specific binding, and false-positive results. In addition, cross-reactivity with closely related circoviruses such as goose circovirus and swan circovirus, caused by partial sequence homology, further undermines diagnostic specificity (1, 19, 20).

Virus-like particles (VLPs) are hollow nanostructures self-assembled from viral capsid subunits that preserve conformational epitopes and mimic native viral morphology (21, 22). Compared with monomeric Cap proteins, VLPs display epitopes in a multivalent and repetitive manner, enabling stronger antigen–antibody interactions and enhanced immunogenicity (23). Thus, VLP-based antigens are expected to substantially improve the specificity, sensitivity, and reliability of serological assays while overcoming the limitations of monomer-based ELISAs (24–26).

In this study, we developed and optimized a VLP-based indirect ELISA (iELISA) for DuCV antibody detection. The full-length Cap of DuCV-2 was efficiently expressed and purified in *E. coli*, and the refolded protein self-assembled into VLPs *in vitro*. These VLPs were used as coating antigens to establish a sensitive, specific, and reproducible iELISA, which was subsequently validated using clinical samples. The developed assay provides a powerful tool for rapid serodiagnosis and epidemiological monitoring of DuCV, and lays a technical foundation for evaluating vaccine efficacy and improving DuCV control strategies.

## 2 Materials and methods

### 2.1 Animals and sample collection

Two categories of serum samples were used in this study: clinical sera for method establishment and validation, and immune sera for immunogenicity evaluation. Clinical serum samples were collected from 10 commercial meat duck farms (Cherry Valley ducks, *n* = 210; local breed ducks, *n* = 80) located in six regions of Jiangsu Province, China (including Xuzhou, Suqian, and Yangzhou) between 2022 and 2024. Ducks were categorized by age: 1–4 weeks (*n* = 105), 5–8 weeks (*n* = 123), and >8 weeks (*n* = 62). Production types included intensive large-scale farms (≥10,000 ducks/farm, *n* = 4 farms, 165 samples) and small-scale backyard farms (<5,000 ducks/farm, *n* = 6 farms, 125 samples). Health status was recorded at sampling: clinically healthy ducks (*n* = 238) and ducks with mild clinical signs (e.g., feather loss, growth retardation, *n* = 52). Sampling was conducted using a stratified random method: within each farm, ducks were randomly selected from different pens (3–5 pens per farm), with sample size proportional to flock size (10–30 samples per farm). Inclusion criteria: ducks with no recent vaccination against DuCV (within 3 months) and no history of severe infectious diseases (e.g., duck enteritis, hepatitis) in the flock within 2 weeks before sampling. Exclusion criteria: ducks with obvious trauma, systemic inflammation, or incomplete medical records. All samples were transported to the laboratory on ice within 24 h and stored at −20 °C until analysis.

Additionally, laboratory-preserved positive sera against duck enteritis virus (DEV), goose parvovirus (GPV), duck tembusu virus (DTMUV), duck hepatitis virus (DHV), goose circovirus (GoCV) and duck-derived bacteria (*riemerella anatipestifer* RA, *salmonella enteritidis* SE, avian pathogenic *Escherichia coli* APEC) were included to verify the specificity of the iELISA.

Immune sera were prepared by immunizing specific-pathogen-free (SPF) ducks (Health-Tech Laboratory Animal Breeding Co., Shandong, China). A total of 25 healthy 20-day-old SPF ducks were randomly divided into five groups (*n* = 5) with similar body weights (≤10% variation). Ducks were immunized subcutaneously in the neck. Two groups received low-dose (0.5 μg) and high-dose (2 μg) recombinant DuCV Cap protein, respectively; two groups received the same doses of DuCV VLPs; and the remaining group received phosphate-buffered saline (PBS) as a negative control. Two weeks after immunization, 2 mL of blood was collected from the wing vein of each duck. Sera were obtained by centrifugation (3,000 rpm, 5 min) after standing at 4 °C, aliquoted, and stored at −20 °C until analysis. These samples were used to compare the immunogenicity of Cap and VLPs.

### 2.2 Expression of Cap protein

The ORF2 gene encoding DuCV Cap (GenBank ID: EU344805, genotype 2a) was codon-optimized for expression in *Escherichia coli* and synthesized commercially (Sangon Biotech, Shanghai, China). The optimized gene was cloned into the pCold I vector (Addgene, MA, United States) and transformed into BL21 (DE3) competent cells (Solarbio, Beijing, China). A single colony was inoculated into 5 mL LB medium containing 100 μg/mL ampicillin and cultured overnight at 37 °C with shaking (220 rpm) as a seed culture. The seed culture was then diluted 1:100 into 200 mL LB medium (100 μg/mL ampicillin) and cultured at 37 °C (220 rpm) until OD₆₀₀ reached 0.8 (approximately 3.5 h). Protein expression was induced by adding 0.5 mM isopropyl-*β*-D-thiogalactoside (IPTG), followed by incubation at 16 °C for 24 h with gentle shaking (180 rpm). Protein solubility was analyzed using SDS-PAGE and Western blotting.

### 2.3 Purification of Cap and assembly of VLPs

Bacterial pellets were harvested by centrifugation (8,000 × g, 10 min, 4 °C) and resuspended in 30 mL lysis buffer (0.1 M NaH₂PO₄, 20 mM imidazole, 300 mM NaCl, 8 M urea, 0.5% Triton X-100, pH 8.0). After sonication (300 W, 5 s on/5 s off, 30 min) and overnight incubation at 4 °C, lysates were ultracentrifuged (100,000 × g, 30 min, 4 °C) to remove debris. The supernatant was loaded onto a 5 mL Ni Sepharose Fast Flow column (GE Healthcare, IL, United States) pre-equilibrated with lysis buffer. The column was washed with 10 column volumes (CV) of wash buffer (0.1 M NaH₂PO₄, 50 mM imidazole, 300 mM NaCl, 8 M urea, pH 8.0) to remove non-specifically bound proteins. The target protein was eluted with 5 CV of elution buffer (0.1 M NaH₂PO₄, 500 mM imidazole, 300 mM NaCl, 8 M urea, pH 8.0). Protein renaturation was performed by stepwise dialysis in buffer (20 mM NaH₂PO₄, 300 mM NaCl, 2 mM *β*-mercaptoethanol, 0.4% arginine, 10% glycerol, pH 7.5), gradually reducing urea concentrations (6 M → 4 M → 2 M → 0 M). Finally, the renatured protein was dialyzed in 0.2 M NaH₂PO₄–Na₂HPO₄ buffer (pH 6.0, 4 °C, 24 h) to induce self-assembly into VLPs.

### 2.4 SDS-PAGE and western blotting

Protein samples were mixed with 5 × loading buffer, boiled for 10 min, and separated on 12% SDS-PAGE gels. Gels were either stained with Coomassie Brilliant Blue R-250 or transferred onto PVDF membranes (Millipore, MA, United States). Membranes were blocked with 5% skim milk for 1 h at room temperature, incubated with anti-His primary antibody (1,5,000, Solarbio), followed by HRP-conjugated goat anti-mouse secondary antibody (1,10,000, Solarbio). After washing with PBST, membranes were developed using enhanced chemiluminescence (ECL) reagent (Millipore).

### 2.5 Transmission electron microscopy

A 10 μL aliquot of purified VLPs was applied to a 200-mesh copper grid for 10 min at room temperature. Excess liquid was removed with filter paper, and the grid was negatively stained with 2% phosphotungstic acid (Solarbio) for 1 min in the dark. After air-drying, samples were examined using a transmission electron microscope (JEM-2100F, JEOL, Tokyo, Japan).

### 2.6 Establishment and optimization of iELISA

VLPs were diluted in 50 mM carbonate buffer (pH 9.6) to varying concentrations, and 100 μL per well was coated onto 96-well microplates (Corning, NY, United States) overnight at 4 °C. For the immunogenicity test, Cap or VLPs were coated at 200 ng/well. After coating, plates were washed three times with PBST and blocked with 0.2% bovine serum albumin (BSA) at 37 °C for 60 min. Serum samples were diluted according to the experimental design (typically 1:200) and incubated at 37 °C for 20 min. Plates were then washed three times, incubated with HRP-labeled mouse anti-duck IgY (1:5000, Solarbio) for 20 min at 37 °C, and washed again. The color reaction was developed using 50 μL TMB substrate at 37 °C for 5 min in the dark and stopped with 50 μL of 2 M H₂SO₄. Each assay included blank, negative, and positive controls. For iELISA optimization, checkerboard titration was performed with three technical replicates per condition. Coating concentrations (0.5–10 μg/mL) and serum dilutions (1,100, 1:150, 1:200) were tested, with each combination assayed in triplicate to calculate mean OD₄₅₀ values and P/N ratios (positive serum OD₄₅₀ / negative serum OD₄₅₀).

### 2.7 Determination of the cut-off value

Forty-five DuCV negative sera were used to determine the ELISA cut-off value. Each sample was tested in triplicate. Each serum sample was tested in three technical replicates, and the signal-to-positive (S/P) ratio was calculated as (sample OD₄₅₀ – blank OD₄₅₀)/(positive control OD₄₅₀ – blank OD₄₅₀). The cut-off value was statistically defined as the mean S/P ratio of negative samples plus 3 standard deviations (SD). Samples with S/*p* values above the cut-off were considered positive, and those below were negative.

### 2.8 Performance evaluation of iELISA

The gold standard for diagnostic performance validation is strictly defined as DuCV-specific real-time quantitative PCR (qPCR) results targeting the conserved region of the DuCV ORF2 gene. A positive gold standard is defined as a qPCR Ct value < 38 with a single melting curve (no non-specific peaks), while a negative gold standard refers to a Ct value ≥ 40 or no amplification curve, with corresponding samples derived from clinically healthy duck flocks confirmed free of DuCV infection via epidemiological surveys.

To assess sensitivity, six positive sera of different reactivity levels were serially diluted (1:100–1:6400) in PBST and tested. The P/N ratio was calculated from OD₄₅₀ values. For specificity testing, positive sera against DEV, GPV, DTMUV, DHV, RA, SE, APEC, and GoCV were diluted 1:200 and analyzed using the optimized protocol. For repeatability and reproducibility, five DuCV positive sera were tested in triplicate within a single plate (intra-assay) and across different plates (inter-assay). The mean S/*p* value, SD, and coefficient of variation (CV = SD/Mean) were calculated. When CV < 10% was considered acceptable.

### 2.9 Statistical analysis

All statistical analyses were performed using SPSS 26.0 (IBM Corp., NY, United States) and GraphPad Prism 9.0 (GraphPad Software, CA, USA). Differences in antibody titers between Cap and VLP immunization groups were analyzed using two-way ANOVA. Intra-assay and inter-assay coefficients of variation (CV) were calculated as (SD/mean) × 100%. Temporal trends in seroprevalence (2022–2024) were analyzed using the Cochran-Armitage test for trend. A significance threshold of *p* < 0.05 was used for all statistical tests.

### 2.10 Ethics statement

All animal experiments were conducted in accordance with the Guide for the Care and Use of Animals in Research of the People’s Republic of China. The study protocol was approved by the Committee on the Ethics of Animal Experiments of Jiangsu Agri-Animal Husbandry Vocational College (Approval No. JSAHVC-2023-35).

## 3 Results

### 3.1 Expression of DuCV Cap and preparation of VLPs

**Figure 1:**
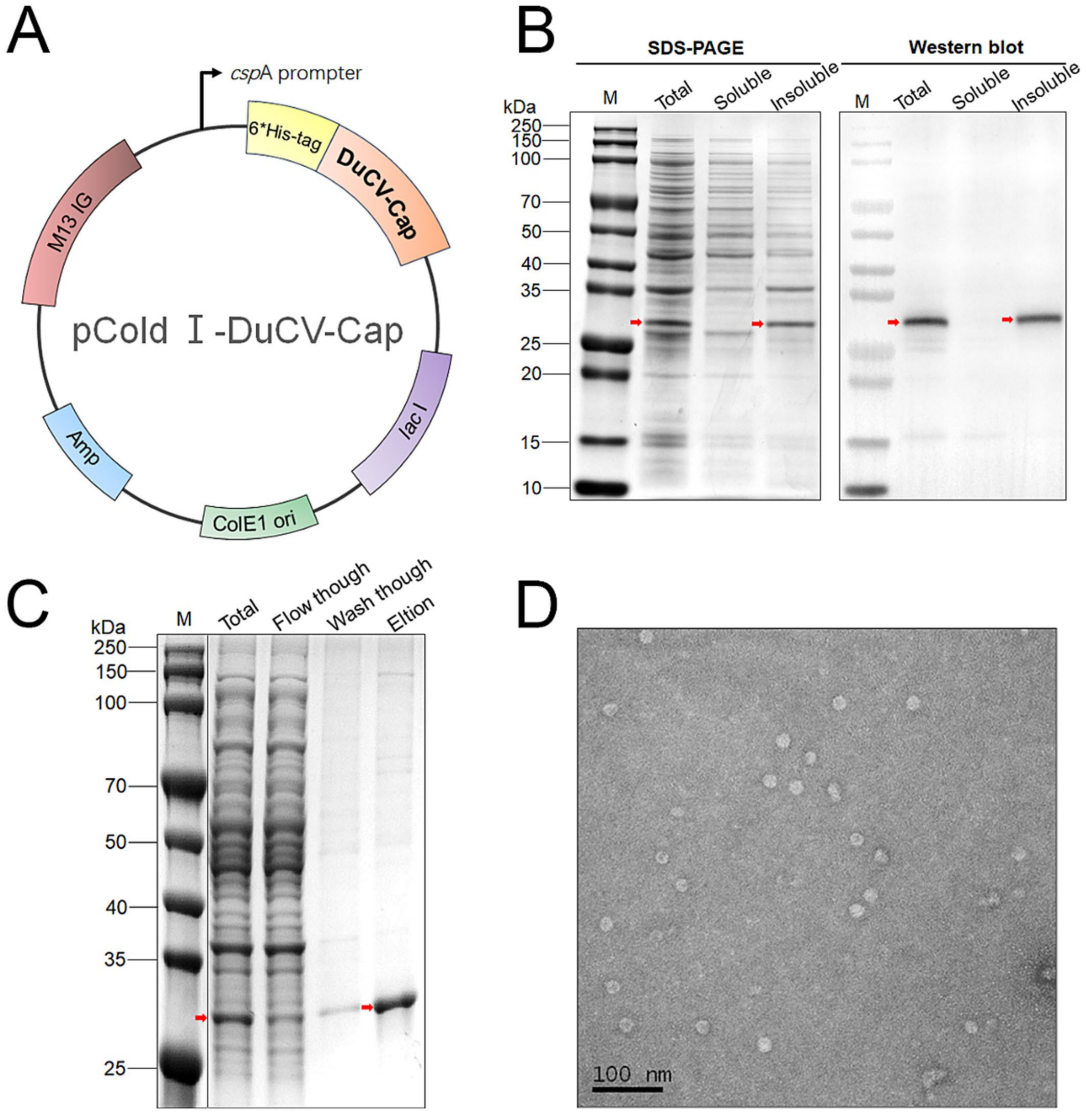
Preparation of DuCV Cap and VLPs. **(A)** Plasmid map of recombinant pCold I-DuCV-Cap. **(B)** Expression and solubility analysis of Cap. **(C)** Denaturing purification of Cap. **(D)** VLPs assembled from Cap observed via TEM.

The full-length DuCV Cap gene was codon-optimized, synthesized, and cloned into the pCold I vector (Figure 1A). Following transformation into *E. coli* BL21 (DE3) cells, protein expression was induced at low temperature with IPTG. SDS-PAGE analysis revealed a distinct band at approximately 30 kDa, corresponding to the theoretical molecular weight of DuCV Cap (Figure 1B). However, the Cap protein accounted for only about 5% of total cellular protein and was predominantly insoluble.

The target protein was subsequently purified under denaturing conditions (8 M urea) using Ni-affinity chromatography (Figure 1C). After gradual refolding by stepwise dialysis, the purified Cap self-assembled into VLPs. Transmission electron microscopy (TEM) revealed numerous spherical particles approximately 15 nm in diameter, consistent with the morphology and size of native DuCV virions (Figure 1D). These observations confirmed that DuCV Cap successfully self-assembled into VLPs *in vitro*.

### 3.2 Immunogenicity of DuCV VLPs

**Figure 2:**
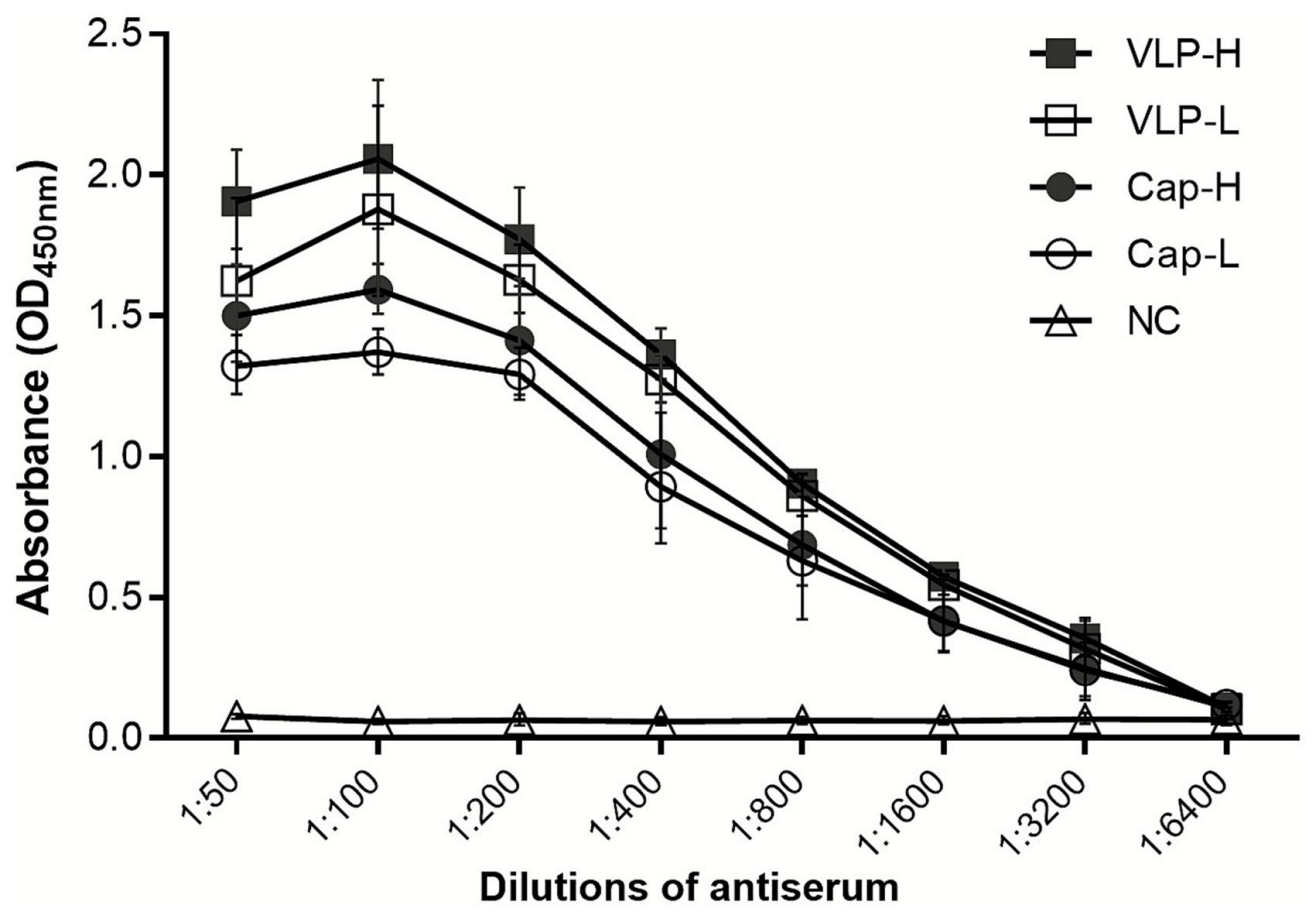
Determination of antibody titers in immunized ducks. Ducks were immunized with low-dose and high-dose Cap or VLPs, respectively. Two weeks later, blood was collected to prepare sera, and the serum antibody titers were determined using the iELISA established with Cap or VLPs.

To compare the immunogenicity of Cap monomers and VLPs, ducks were immunized with low (0.5 μg) or high (2 μg) doses of each antigen. Serum samples were collected 2 weeks after immunization and analyzed by ELISA. Both Cap and VLP groups elicited specific antibody responses, whereas the PBS control group remained negative (Figure 2). At equivalent doses, VLPs induced significantly higher antibody titers than Cap monomers. Notably, even the low-dose VLP group produced higher antibody levels than both Cap groups, indicating that VLPs possessed markedly superior immunogenicity.

### 3.3 Establishment and optimization of the iELISA

The positive-to-negative (P/N) ratio was used to determine the optimal antigen coating concentration and serum dilution by checkerboard titration. As shown in Table 1, the highest P/N ratio was obtained when the coating buffer was 50 mM carbonate–bicarbonate (pH 9.6), the antigen concentration was 2.5 μg/mL (equivalent to 250 ng/well), and the serum dilution was 1:200.

**Table 1:** Checker board titration of the DuCV VLP.

Serum dilution	Concentration of coating antigen (X ± SD, μg/ml)					
	0.5	1	2.5	5	7.5	10
100 × (+)	0.843 ± 0.023	1.17 ± 0.019	1.348 ± 0.061	1.756 ± 0.037	2.122 ± 0.037	2.116 ± 0.029
100 × (−)	0.098 ± 0.036	0.099 ± 0.055	0.122 ± 0.065	0.147 ± 0.044	0.188 ± 0.028	0.206 ± 0.034
P/N	8.608	11.705	11.020	11.878	11.250	10.274
150 × (+)	0.854 ± 0.065	0.958 ± 0.044	1.298 ± 0.084	1.684 ± 0.051	1.815 ± 0.033	2.1 ± 0.051
150 × (−)	0.082 ± 0.086	0.09 ± 0.066	0.115 ± 0.074	0.135 ± 0.056	0.165 ± 0.041	0.198 ± 0.042
P/N	10.341	10.562	11.265	12.438	10.988	10.558
200 × (+)	0.63 ± 0.045	0.802 ± 0.078	1.205 ± 0.023	1.453 ± 0.026	1.819 ± 0.036	2.005 ± 0.039
200 × (−)	0.084 ± 0.031	0.077 ± 0.100	0.093 ± 0.046	0.114 ± 0.045	0.153 ± 0.018	0.196 ± 0.056
P/N	7.442	10.356	12.959	12.721	11.890	10.185

Other assay parameters were optimized to further enhance detection performance. The final optimized conditions were as follows: coating overnight at 4 °C; blocking with 0.2% bovine serum albumin (BSA) at 37 °C for 60 min; using HRP-conjugated mouse anti-duck IgY (1:5000) as the secondary antibody; and color development with TMB substrate for 5 min at 37 °C in the dark. The reaction was terminated with 2 M H₂SO₄, and the absorbance at 450 nm (OD₄₅₀) was recorded. This protocol ensured stable color development and reproducible signal intensity.

### 3.4 Determination of the iELISA cut-off value

**Figure 3:**
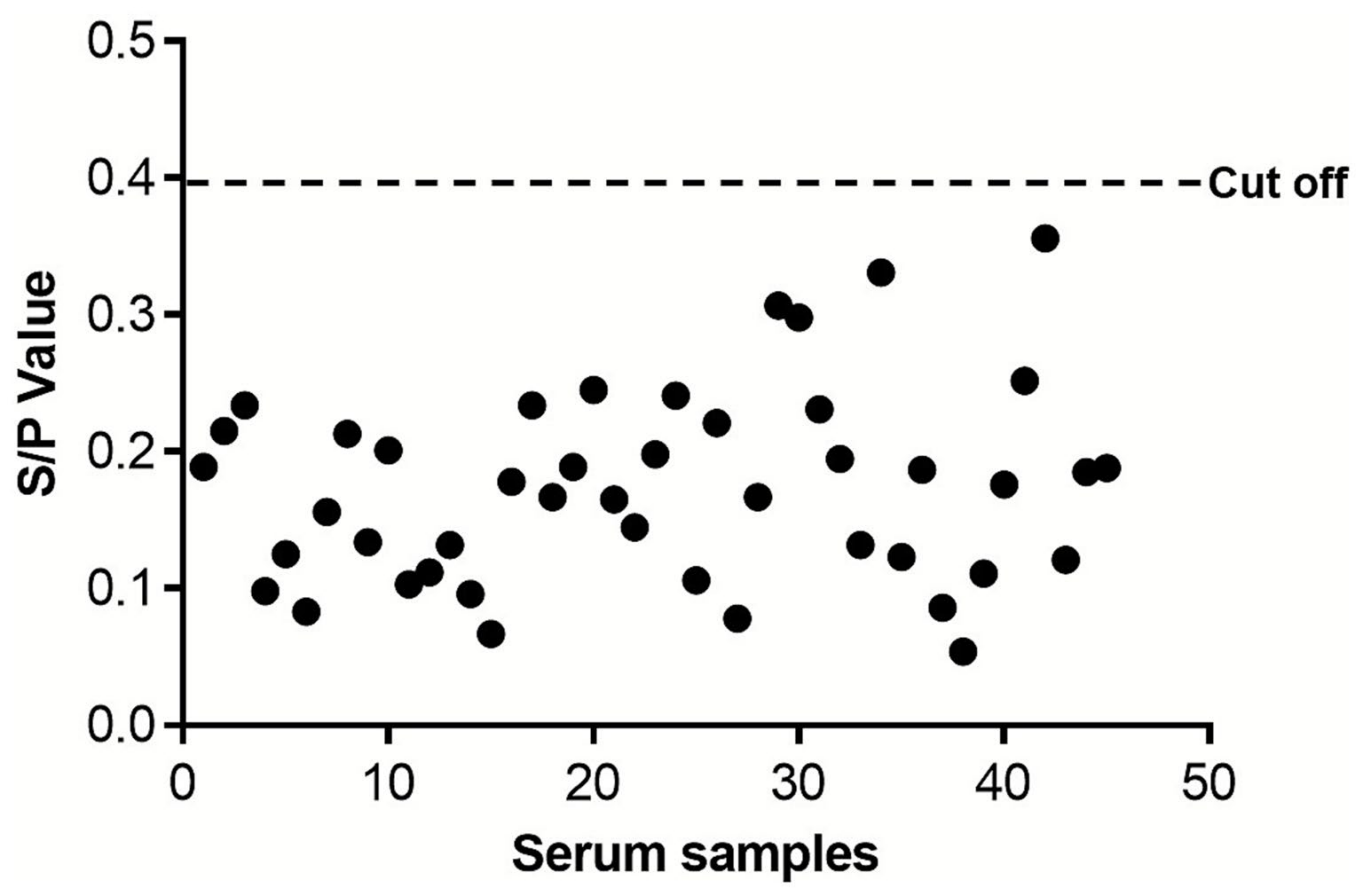
Evaluation of the cut-off value of the indirect ELISA. Each black dot represents the S/*p* value of an individual DuCV-negative serum sample (*n* = 45). The red horizontal line indicates the cut-off value (0.396) calculated as the mean S/P ratio of negative samples plus 3 standard deviations.

Forty-five negative serum samples were used to determine the cut-off value of the established ELISA. All serum samples were tested in triplicate to minimize experimental bias. After measuring the OD₄₅₀ values, the mean signal-to-positive (S/P) ratio of all negative samples detected by the DuCV VLP-based ELISA was calculated as 0.156, with a standard deviation (SD) of 0.080 (Figure 3). Accordingly, the cut-off value of this ELISA was defined as 0.396, which was derived from the formula: mean S/P ratio + 3 × SD. Samples with an S/P ratio higher than this cut-off value were classified as positive, while those with an S/P ratio lower than this value were classified as negative.

### 3.5 Performance evaluation of the VLP-iELISA

**Figure 4:**
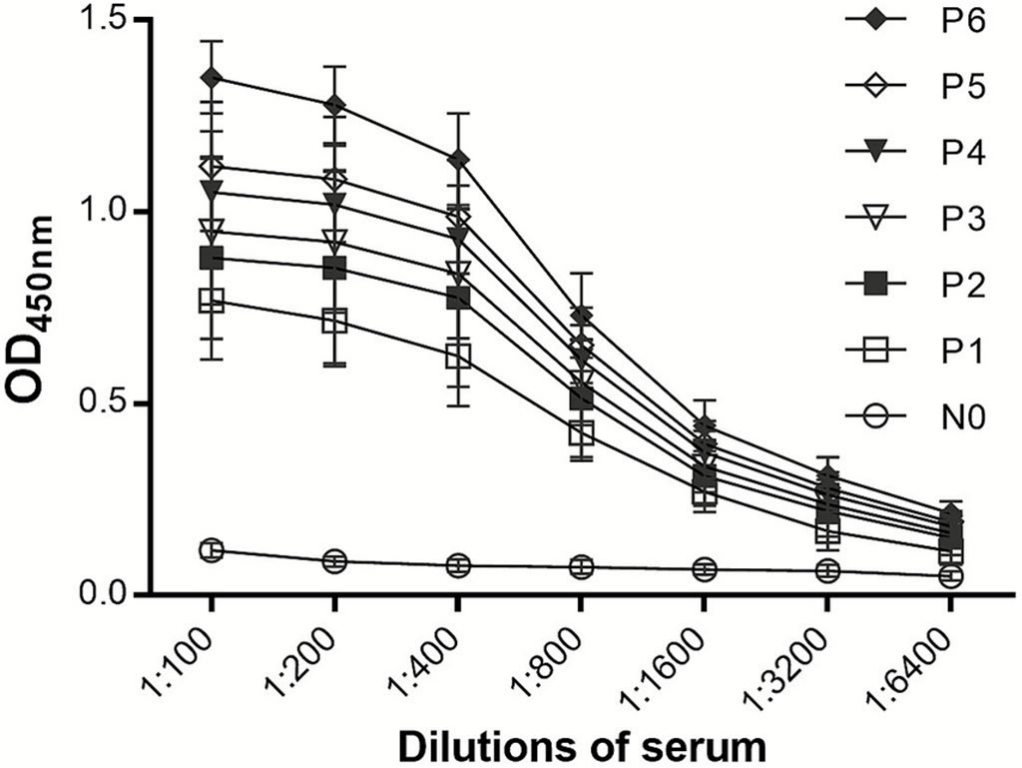
Determination of the sensitivity of iELISA.

Six DuCV positive sera with varying reactivity were serially diluted twofold (1:100–1:6400) and tested by the established iELISA. As shown in Figure 4, positive signals were detectable up to a 1:6400 dilution, demonstrating the high sensitivity of the VLP-based assay.

To evaluate assay specificity, positive sera against DEV, GPV, DTMUV, DHV, RA, SE, APEC, and GoCV were tested at a 1:200 dilution (Table 2). The VLP-based iELISA showed no cross-reactivity with any of these pathogens. In contrast, the Cap-based iELISA produced a false-positive reaction with GoCV-positive serum, likely due to partial sequence homology between DuCV and GoCV Cap proteins. These results confirmed that VLPs preserved conformational epitopes unique to DuCV, thereby improving assay specificity.

**Table 2:** Comparison the specificity of DuCV Cap-iELISA and VLP-iELISA.

Pathogen	qPCR of DuCV	Cap-iELISA (OD₄₅₀)	VLP-iELISA (OD₄₅₀)
DuCV	Positve	**0.986 ± 0.065**	**0.889 ± 0.028**
duck Enteritis Virus (DEV)	Negative	0.176 ± 0.035	0.087 ± 0.038
goose parvovirus (GPV)	Negative	0.135 ± 0.046	0.105 ± 0.021
duck tembusu virus (DTMUV)	Negative	0.196 ± 0.031	0.089 ± 0.033
duck hepatitis virus (DHV)	Negative	0.096 ± 0.040	0.083 ± 0.055
goose circovirus (GoCV)	Negative	**0.590 ± 0.153**	0.130 ± 0.063
*Riemerella anatipestifer* (RA)	Negative	0.101 ± 0.026	0.082 ± 0.026
*Salmonella* Enteritidis (SE)	Negative	0.087 ± 0.032	0.077 ± 0.037
avian pathogenic *E.coli* (APEC)	Negative	0.092 ± 0.035	0.093 ± 0.042

Bold values indicate the OD₄₅₀ values that exceed the cut-off threshold (0.396) of the Cap-iELISA, indicating positive cross-reactivity.

Five DuCV positive sera were selected to assess intra- and inter-assay variation. The intra-assay CV ranged from 2.1 to 3.5%, and the inter-assay CV ranged from 2.9 to 4.8% (Table 3), both well below the 10% threshold. Additionally, based on the 290 qPCR-verified clinical samples (57 positive and 233 negative), the positive predictive value (PPV) and negative predictive value (NPV) of the VLP-iELISA were calculated as 100.0% (95% confidence interval: 93.5–100.0%) and 100.0% (95% confidence interval: 98.4–100.0%), respectively. These results demonstrated that the VLP-based iELISA exhibited excellent repeatability and reliability.

**Table 3:** Repeat ability test of VLP-iELISA.

Sample no.	Intra-assay (S/P)						Inter-assay (S/P)					
	1	2	3	Mean	SD	CV%	1	2	3	Mean	SD	CV%
#1	0.868	0.889	0.912	0.890	0.022	2.5	0.938	0.956	0.890	0.928	0.034	3.7
#2	1.116	1.043	1.065	1.075	0.037	3.5	0.913	1.003	0.947	0.954	0.045	4.8
#3	0.922	0.946	0.974	0.947	0.026	2.7	0.922	0.966	0.974	0.954	0.028	2.9
#4	1.078	1.103	1.125	1.102	0.024	2.1	1.078	1.103	1.025	1.069	0.040	3.7
#5	0.932	0.955	0.973	0.953	0.021	2.2	0.832	0.895	0.873	0.867	0.032	3.7

### 3.6 Detection of clinical samples

A total of 290 clinical serum samples collected from duck farms in Jiangsu Province (2022–2024) were tested using the established VLP-iELISA. As summarized in Tables 4, 57 samples were positive and 233 were negative, corresponding to an overall positivity rate of 19.96%. Descriptively, yearly positivity rates showed a gradual numerical increase: 15.29% (13/85) in 2022, 19.05% (20/105) in 2023, and 24.00% (24/100) in 2024. Statistical analysis using the Cochran-Armitage test for trend confirmed that this temporal increase was not statistically significant (*p* = 0.123). This lack of statistical significance is likely attributed to limited sample size in each year, which reduces the statistical power of the test and limits the ability to detect a true underlying trend. Notably, despite the absence of statistical significance, the consistent numerical upward trend suggests that DuCV infection may be gradually spreading within Jiangsu Province, and this trend warrants verification with expanded sample sizes in future surveillance. Regionally, the highest positivity rate was observed in Yancheng (28.21%), followed by Nantong (25.00%), while Yangzhou (13.95%) had the lowest. These findings suggest that DuCV infection remains prevalent and may be spreading gradually within Jiangsu Province.

**Table 4:** Detection of DuCV specific antibody in sera from meat ducks.

Areas in Jiangsu Province	Positive	Negative	Total	Positive rate (%)
Xuzhou	18	70	88	20.45
Suqian	8	48	56	14.29
Yangzhou	6	37	43	13.95
Huaian	5	23	28	17.86
Yancheng	11	28	39	28.21
Nantong	9	27	36	25.00
Total	57	233	290	19.96

## 4 Discussion

This study aimed to address the limitations of existing DuCV serological assays by developing a VLP-based iELISA with improved sensitivity and specificity. Through systematic optimization, we successfully prepared the recombinant full-length Cap protein of DuCV-2, which self-assembled into uniform VLPs *in vitro*. Using these VLPs as coating antigens, we established an accurate and reproducible diagnostic assay for DuCV antibody detection, providing a valuable tool for both laboratory research and field surveillance.

To date, research on DuCV ELISA detection methods remains relatively limited. Three prior studies, published in 2010, 2014, and 2017 respectively, all utilized the pET-32a(+) vector to express recombinant Cap in *E.coli*. The target antigens were purified via Ni-NTA affinity chromatography, and iELISA assays were subsequently established for detecting DuCV specific antibodies in duck serum (19, 20, 27). A common drawback among these assays is that they rely on monomeric Cap proteins, which are susceptible to misfolding and denaturation, leading to loss of conformational epitopes and cross-reactivity. In contrast, our study is the first to employ VLPs as coating antigens for DuCV serodiagnosis, introducing a novel structural antigen-based strategy for improving assay performance.

While using capsid monomer proteins of non-enveloped viruses as coating antigens is a conventional practice in ELISA development, VLP-based iELISA offers distinct advantages over Cap-based iELISA. From a methodological perspective, the VLP-based iELISA required a lower coating antigen concentration (250 ng/well) compared with previously reported Cap-based assays (400 ng/well and 4 μg/well) (19, 20), which may reduce production costs while maintaining high reactivity. Moreover, the sensitivity of our assay (1:6400) markedly exceeded that of earlier methods (1:256–1:2560), confirming that VLPs significantly enhance detection efficiency. These improvements can be attributed to the structural integrity and high-density epitope presentation of VLPs, which better mimic the native virus and preserve conformational epitopes essential for specific antibody recognition (Table 5).

**Table 5:** The advantages of VLP-based iELISA compared to Cap-based iELISA.

Comparison dimension	VLP-based iELISA	Cap-based iELISA
Antigen Conformation	Mimics native viruses, retains intact conformational epitopes	Prone to denaturation, loses conformational epitopes, only retains linear epitopes
Epitope Density	Multivalent and high-density display	Monovalent and low-density display
Detection Sensitivity	High (capable of detecting low-concentration antibodies)	Low (prone to missing low-concentration antibodies, leading to false negatives)
Detection Specificity	High (no cross-reactivity, low false positive rate)	Low (prone to cross-reactivity, high false positive rate)
Stability	Strong (resistant to degradation, tolerant to temperature and pH)	Weak (prone to denaturation and aggregation)
Batch-to-Batch Consistency	Good (uniform assembly, simple quality control)	Poor (large variation in folding efficiency)
Clinical Relevance	High (directly detects neutralizing antibodies, reflects protective efficacy)	Low (only detects total antibodies, cannot distinguish antibody function)
Anti-Interference Ability	Strong (high-affinity binding, resistant to matrix interference)	Weak (susceptible to interference from interfering substances)

The advantages of VLP-based iELISAs have been well-documented in other viral systems. For example, a swine vesicular disease VLP-based iELISA achieved diagnostic specificities of 98.7% for IgM and 99.6% for IgG, substantially reducing false positive rate compared with traditional neutralization tests (28). Similarly, a SARS-CoV-2 VLP-based iELISA reached a sensitivity of 97.5% and specificity of 100%, outperforming certified *in vitro* diagnostic devices (29). These reports, together with our findings, highlight that VLP-based platforms offer superior diagnostic reliability by preserving native epitope structures and reducing cross-reactivity.

In the present study, we further demonstrated that the Cap-based iELISA exhibited strong cross-reactivity with GoCV positive sera, while the VLP-based iELISA completely eliminated this interference. Considering that GoCV and DuCV Cap proteins share up to 47.1% sequence identity (1), this distinction underscores the diagnostic precision of the VLP approach. Given that swan circovirus and other waterfowl circoviruses (e.g., American wild duck circovirus and WigFec circovirus 1) also exhibit partial sequence homology (27–55%), VLP-based antigens are expected to minimize false positive rate even in regions where multiple circoviruses co-circulate.

The increase in DuCV seroprevalence from 2022 to 2024 may be attributed to several plausible factors. First, the expansion of intensive duck farming in Jiangsu Province has increased duck density, facilitating viral transmission between flocks. Second, improved awareness of DuCV among farmers may have reduced underreporting of subclinical infections, although this does not directly explain increased seroprevalence. Third, co-infections with other immunosuppressive pathogens (e.g., novel goose parvovirus, NGPV; fowl adenovirus) may enhance DuCV replication and spread. However, without data on farm management practices (e.g., biosecurity measures, flock turnover) or vaccination status, these explanations remain speculative.

Despite these promising results, certain limitations should be acknowledged. First, the sample size and geographic coverage were relatively limited, which may restrict the generalizability of the epidemiological conclusions. Future studies should expand surveillance to additional provinces and larger flocks to better evaluate the assay’s applicability. Second, as this study utilized VLPs derived from DuCV-2, the ability of the assay to detect antibodies against other DuCV genotypes (DuCV-1 and DuCV-3) remains to be determined. Future validation should include sera from ducks experimentally infected with DuCV-1 and DuCV-3, as well as field samples from regions with known circulation of these genotypes. Additionally, the development of chimeric VLPs incorporating conserved epitopes from all three genotypes could enhance the assay’s broad-spectrum utility. Third, the specificity test could not include some relevant co-infecting viruses, such as novel goose parvovirus (NGPV), which frequently co-occurs with DuCV in clinical settings and causes “beak atrophy dwarfism syndrome” in ducks (30). Addressing these issues will further enhance the diagnostic utility of this assay.

The VLP-iELISA developed in this study offers practical benefits for duck farm management and regional surveillance programs. Its high sensitivity enables early detection of subclinical infections, allowing farmers to implement timely biosecurity measures to prevent viral spread. The low coating concentration reduces reagent costs, making it suitable for large-scale screening of commercial flocks. For epidemiological surveillance, the assay’s specificity eliminates false positives from co-circulating circoviruses (e.g., GoCV), ensuring accurate estimation of DuCV prevalence. Additionally, the assay can be used to evaluate vaccine efficacy by monitoring antibody titers post-vaccination, supporting the development of targeted control strategies. At the farm level, routine testing with the VLP-iELISA can inform breeding decisions and reduce economic losses associated with secondary infections.

Collectively, the DuCV VLP-based iELISA developed in this study provides a powerful and practical serological tool for large-scale epidemiological surveys and vaccine evaluation. By accurately detecting antibodies against DuCV, this assay enables timely monitoring of infection dynamics and supports evidence-based prevention strategies in duck farms. Moreover, the prokaryotic VLP production strategy demonstrated here offers a cost-effective and adaptable framework for developing diagnostic assays against other avian circoviruses. This approach not only promotes the advancement of veterinary diagnostic technology but also contributes to the broader understanding and control of circovirus-associated diseases in the poultry industry.

## Data Availability

The original contributions presented in the study are included in the article/supplementary material, further inquiries can be directed to the corresponding author.
